# Irrational Consumption during the COVID-19 Period

**DOI:** 10.3390/ijerph19095031

**Published:** 2022-04-21

**Authors:** Wenhuan Yu, Lin He, Xianhao Lin, Thomas Freudenreich, Tao Liu

**Affiliations:** 1 School of Management, Zhejiang University, Hangzhou 310058, China; 11820032@zju.edu.cn (W.Y.); helin@zju.edu.cn (L.H.); thomas.freudenreich@wu.ac.at (T.F.); 2 School of Management, Shanghai University, Shanghai 200444, China; 3 School of Health, Fujian Medical University, Fuzhou 350122, China; linxh@fjmu.edu.cn; 4 Institute for International Marketing Management, Vienna University of Economics and Business, 1020 Vienna, Austria

**Keywords:** COVID-19, irrational consumption, stress, sleep quality, negative emotion

## Abstract

The outbreak of the COVID-19 pandemic has severely impacted the world economy and has, most presumably, exerted a great deal of stress on citizens, in turn leading to the call for timely assessments of how this period might actually impact individuals at the level of everyday well-being and in their behaviors such as consumer decisions. Through one pilot study and two online survey studies, we tentatively investigated this latter question, and demonstrated that the COVID-19 pandemic may increase perceived stress and impair individuals’ sleep quality, which in turn impels their irrational consumption. This research provides preliminary evidence for the impact of the present pandemic on irrational consumption and contributes to the literature on stress and consumer behavior.

## 1. Introduction

The outbreak of the COVID-19 pandemic has affected the lives of billions worldwide. In addition to causing economic upheaval and political unrest, the pandemic poses a major challenge to physical and mental public health. Specifically, governments across the world have enacted strict control measures as a means of containing the viral spread and have fundamentally disrupted typical life activities and social interactions. Not surprisingly, these changes have shown an additional strain on people’s psyche in the form of increased stress or anxiety [1,2,3,4]. In turn, a global call for timely research on this topic, such as the actual quantification of impacts and individual coping strategies or behavioral responses, has been demanded.

One activity that has been particularly affected by the COVID-19 pandemic is shopping. Emerging evidence illustrates that, during the pandemic, consumers shopped more online, made more bulk purchases, and relied more on delivery methods for food or other consumables [5,6,7]. Previous studies have suggested that increased anxiety or stress may prompt consumers to make more impulsive purchases [8], as a means of coping or “retail therapy” [5]—seeking out temporary hits of pleasure and/or control over at least one life aspect. While being potentially relieving, increased shopping can also be problematic, leading to more impulsive and less rational purchases [8]. This behavior is associated with irrational consumption, which can be defined as unreasonable hoard of particular goods, and excessive consumption [6,7], eventually causing supply shortages and overall financial distress. Thus, irrational consumption becomes an important factor to be considered not only to better understand our everyday behavior, which is currently affected by COVID-19, but also to further expand our understanding of consumer behavior in prolonged stressful environments in general.

This raises the questions of whether irrational consumption might be found during the ongoing COVID-19 situation; perhaps more importantly, how such shopping patterns might relate to actual stress; and what are the underlying mechanisms. In the present research, we consider individuals’ behaviors of irrational consumption during the COVID-19 pandemic to examine the effect of perceived stress induced by the pandemic and to uncover its impacts on the individual and wider consumer space as well as its underlying mechanisms.

We carry this out through a pilot study and two online survey studies. The pilot study aimed to provide preliminarily evidence that the COVID-19 pandemic was linked to individuals’ irrational consumption using second-hand data obtained from the official websites of the State Post Bureau of The People’s Republic of China and the National Bureau of Statistics. The purpose of study 1 was twofold. First, it was designed to examine whether the severity of the COVID-19 pandemic would increase individuals’ perceived stress and further lead to irrational consumption. Second, the aim was to investigate the impact of stress on irrational consumption, revealing the potential mediating role of sleep quality. That is, the COVID-19 pandemic may exert additional stress, which impairs individuals’ sleep quality and, in turn, might induce irrational consumption. Study 2 was then designed to replicate the impact of the COVID-19 severity on perceived stress and irrational consumption as well as the mediating role of sleep quality among consumers with different cultural background.

As such, this is geared to both give an important window into the current situation and to contribute to the literature of abnormal consumption in stressful environments. Below, we briefly review the study’s main aspects—stress, irrational shopping, and sleep quality—as well as our hypotheses before moving on to the empirical studies.

## 2. Theoretical Background

The present study had, at its core, a rather basic hypothesis that we would find a general increase in perceived stress with escalation of the severity of the COVID-19 pandemic as well as a concomitant increase in irrational consumption.

### 2.1. Perceived Stress and Irrational Buying

Perceived stress refers to individuals’ subjective feelings on internal or external stressful events. Lazarus and Folkman [9] proposed that, when demand exceeds one’s ability or resources, it can cause psychological stress. Similar factors are also reflected in subjective stress assessment scales, such as the Perceived Stress Scale [10], used in the present study, which includes items involving, for instance, unpredictability and uncontrollability in daily-life as well as a sense of overloading. Such responses are again clearly found in the current pandemic, and ample studies have documented the rising mental stress induced by the COVID-19 pandemic, which has been menacing the psychological wellbeing of people [11].

In response to mental stress, research suggests that individuals may engage in various coping activities. For instance, seeking out exercise and social engagements, or performing other enjoyable hobbies might be appropriate solutions; the control measures, preventing the spread of the Coronavirus, however, made these impossible [12]. In turn, people may engage in unhealthy or irrational activities to cope with the stress, such as excessive drinking [13], seeking out snack foods [14], or impulsive shopping [5,15,16]. Previous research has also shown particular increases in impulsive buying during widespread events such as natural disasters [5,6,7].

Based on these findings, we hypothesized that the COVID-19 pandemic would increase individuals’ perceived stress, which in turn would induce irrational consumption.

### 2.2. Perceived Stress, Negative Emotions, and Irrational Consumption

Perceived stress affects both individuals’ physical and emotional well-being. On one hand, prolonged stress is related to increased illnesses, including cancer, diabetes, cardiovascular disease, asthma, and rheumatoid arthritis [17]. Even short spikes in stress may impact diurnal rhythms, cortisol secretion, or other body processes [18,19,20]. On the other hand, perceived stress may also lead to negative thoughts or emotions [21,22]. For example, a large survey of 1275 subjects showed significant positive relations between perceived stress and anxiety as well as depression, which was further associated with suicidal ideation [23]. In the same vein, evidence from a wide range of studies has reported that perceived stress is closely associated with individuals’ negative emotions and anxiety disorder [24].

Consumption has been confirmed to be a coping strategy to mitigate negative emotions, especially while faced with severe stressful events [25]. For example, Kemp and Williams [26] proposed that, in the process of preparing for an impending catastrophe, individuals may engage in emotion-focused coping by engaging in consumption to make efforts to mitigate negative emotional outcomes. Mick and colleagues [27] also identified that people often give gifts to themselves to cope with disappointments and depression.

Thus, we hypothesized that individuals in the COVID-19 period may experience enhanced stress, inducing negative emotion, which would finally lead to irrational consumption.

### 2.3. Perceived Stress, Sleep Quality, and Irrational Behavior

Besides negative emotions, we also considered sleep quality as a potentially important mediator in the prolonged COVID-19 pandemic from a long-term perspective.

Sleep problems have become serious issues across the globe during the COVID-19 pandemic. The most typical symptoms include poor quality, longer time taken to fall asleep, increased discontinuous sleep, and sleeping pills consumption [28,29]. In a worldwide survey study, 58% of the respondents (2562) from 49 countries were unsatisfied with their sleep quality compared with that before the COVID-19 crisis [28]. More specifically, recent studies have demonstrated that, compared to pre-COVID-19 times, sleep quality was reported to be significantly worse for respondents from Australia [30], Bangladesh [31], China [32], Israel [33], the United States [33,34], and Turkey [35].

The reason for these severe sleep problems is that the COVID-19 pandemic has brought an unprecedented number of uncontrollable stressors [36] including financial stress, supply and health-related stress, as well as social isolation [37]. Coiro et al. [33] revealed that the respondents who reported more COVID-related stress also reported significantly poorer sleep quality. Similarly, Werner et al. [38] reported that the increased stress induced by the COVID-19 pandemic may affect individuals’ sleep quality, leading to negative changes in overall sleep and pre-sleep arousal.

This line of research is consistent with previous literature focusing on the relationship between stress and sleep. Past research demonstrated that individuals suffering from long-term stress may report being exhausted or having sleep problems [39]. Theadom and Cropley [40] examined the relation between perceived stress and sleep quality in patients with fibromyalgia and confirmed that perceived stress was significantly associated with sleep disorder. Brand et al. [41] and Kashani et al. [42] have also revealed the same stress–sleep-quality relation in both healthy adolescents (5580 samples) and patients with cardiovascular disease.

Overall, it can be said that sleep is critical for both physical and mental health and that poor quality of sleep can lead to increased risk-taking and impulsive behavior [43]. Previous neuroscience research has shown that sleep deprivation may undermine cognitive functions, especially the higher-order functions involved in the prefrontal cortex, which are associated with risk-taking and impulsive behavior, such as executive function, self-control, and action inhibition [44,45,46,47]. For instance, Jugovac and Cavallero [43] demonstrated that, after 24 h of sleep deprivation, the participants’ attention became distracted, and their executive control efficacy decreased significantly, which increased subsequent risk-taking behavior. Behavioral studies have also provided evidence on it. Demos et al. [48], using Go/No-Go and other impulsive-behavior paradigms, found that even partial sleep deprivation increased the possibility to perform irrational behaviors. In the context of the COVID-19 pandemic, recent studies have again demonstrated that poorer sleep quality during the COVID-19 pandemic may result in higher daily dysfunctions [29].

Since previous studies have consistently reported that perceived stress may undermine sleep quality and, in turn, may induce irrational behavior, we hypothesized that sleep quality would mediate stress-driven irrational consumption during the COVID-19 period. Specifically, the level of perceived stress would correlate with individuals’ sleep quality and the level of sleep quality would show a negative relation with irrational consumption.

It is noteworthy here that most previous studies showing a relation between stress and sleep quality have mainly adopted paradigms of sleep deprivation and focused on participants’ irrational behaviors in psychological tasks within laboratories. The present study may offer a more natural way of observing consumer behavior and sleep quality during prolonged stressful events.

## 3. Pilot Study

This pilot study aimed to provide preliminarily evidence that the COVID-19 pandemic may affect individuals’ consumption behavior which, in turn, is linked to irrational consumption in particular. To address this issue, we collected data of the total inter-city package volume (the number of packages delivered across cities) for each province or municipality (except for Hubei province, because the COVID-19 pandemic is extremely severe in Hubei Province, many express companies closed or delivered anti-pandemic materials only, especially in February, so the number of packages delivered cannot represent individuals’ consumption; N = 30) in the first quarter of 2020 (M ± SD = 412.38 ± 735.41 million) from the official website of State Post Bureau of The People’s Republic of China as well as the corresponding regional Gross Domestic Product (GDP) (CNY 662.61 ± 552.65 billion) and total population (45.28 ± 29.12 million people, at the end of 2019) from the National Bureau of Statistics (data.stats.gov.cn, accessed on 1 February 2021). To control the impact of consumption capacity in the data analysis, we then calculated the per capita GDP for each province or municipality (CNY 14.56 ± 6.87 thousand).

**Figure 1:**
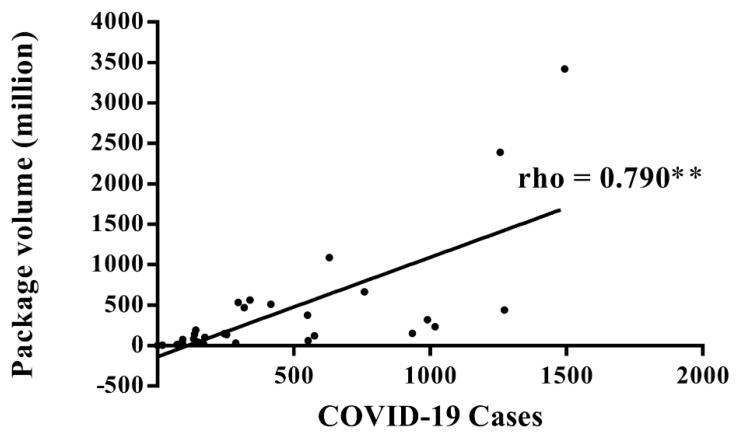
Correlation between the number of COVID-19 cases and the number of packages in the first quarter of 2000 for the 30 provinces or municipalities of China (except for Hubei province). ** represents *p* < 0.001.

The Spearman’s analysis revealed a significant positive correlation between the inter-city package volume and the COVID-19 cases (Rho = 0.790, *p* < 0.01), as shown in Figure 1. The inter-city packet volume also significantly correlated with the total population (Rho = 0.721, *p* < 0.01) and per capital GDP (Rho = 0.645, *p* < 0.01). The hierarchical multiple regression analysis further confirmed that, while taking total population (beta = 0.251, *p* = 0.216) and GDP per capital (beta = 0.327, *p* = 0.019) as covariates, the COVID-19 cases still positively correlated with the inter-city packet volume (adjusted R^2^ = 0.547, F = 12.682, beta = 0.430, *p* = 0.044).

Although inter-city packet volume cannot exactly represent consumption data, it is noteworthy that, in the first quarter of the year 2020, the COVID-19 pandemic in China was the most severe. During this period, the government has implemented strict controls over living necessities for survival, such as meat, vegetables and masks. In this context, these essential living materials were obtained mainly via local delivery service within the cities or would be allocated by local government delivering by volunteers. All these services were not registered in the inter-city packet volume. Except for the essential living materials, almost everything else can only be purchased online. Therefore, the number of packages could be used to depict people’s consumption behavior to a certain extent. After controlling the population and GDP per capita, in the first quarter of 2020, the pandemic severity was positively correlated with the number of packages, indicating that the more severe the pandemic, the more things people tend to buy.

Concerning the items purchased online, according to the data obtained from the Department of Commerce of Zhejiang Province, China (http://www.zcom.gov.cn, accessed on 8 April 2022), in the first quarter of 2020, the online shopping categories in Zhejiang Province mainly covered Clothes, Shoes, and Bags (31.6%); Household items (18.8); Digital products (14.8); Babies and Kids (8.2%); Skincare (7.4%); and Health foods (6.4%).

In order to provide more evidence to the hypothesis that the COVID-19 may lead to an increased shopping tendency, we also analyzed the influencing factors of the inter-city package number in the second quarter of 2020 (688.71 ± 1285.17 million), when the COVID-19 pandemic had been well controlled in China. We subtracted the cumulative number of confirmed cases on March 31 from that of June 30 to obtain the increased number of the confirmed cases in each province during the second quarter of 2020 (66.13 ± 117.95), which served as the indicator of the severity of the COVID-19 pandemic.

The Spearman’s analysis revealed that the inter-city packet volume significantly correlated with the total population (Rho = 0.748, *p* < 0.01), GDP (Rho = 0.935, *p* < 0.01), and GDP per capital (Rho = 0.639, *p* < 0.01). However, the increased number of confirmed cases in each province was no longer correlated with the number of inter-city packages (*p* = 0.082). That is to say, as the severity of the pandemic gradually reduced, the number of inter-city packages in each province were only related to the population and GDP per capita, which was consistent with the periods without the COVID-19 pandemic. This evidence indirectly suggests that, in the first quarter of 2020, the severity of the pandemic prompted people to buy more products, whereas with the decline of the COVID-19 pandemic, individuals’ consumption behaviors gradually returned to normal.

In summary, using secondary data, this pilot study preliminarily confirmed that the COVID-19 pandemic altered individuals’ shopping behavior and may induce increased tendency of irrational shopping. With a surge of severity in the pandemic, the number of products purchased by consumers increased significantly, which may be a typical manifestation of irrational consumption [6]. Past studies have also demonstrated that severe outbreaks of the COVID-19 pandemic may evoke irrational consumption such as panic buying [6,7].

## 4. Study 1

In Study 1, we attempted to directly examine the impact of COVID-19 severity on individuals’ stress level as well as their irrational consumption tendency; 422 Chinese respondents (male = 235, female = 187; age = 28.59 ± 6.84; monthly income = CNY 9532.69 ± 9200.03) were recruited via Credamo, a data collecting and modeling platform in China. The participants represented a range of cities and regions (27 provinces or municipalities; 90 cities), which were differentially impacted by the pandemic during the data collection period.

The power analysis was conducted with the G-power v3.1.9.2 (F. Faul et al.; http://www.gpower.hhu.de, accessed on 8 April 2022). When the effect size was 0.15, the significance level was 0.05, and 7 predictors were arranged; the total sample size should be more than 153. Thus, the sample size used in Study 1 was enough to reveal the relation between the pandemic severity and irrational consumption tendency and to uncover its underlying mechanism, emphasizing the potential mediating roles of stress and sleep quality or negative emotion.

For the measurement of COVID-19 severity, we selected the cumulative number of confirmed cases in each province and city during May 2020 from the website of the National Health Commission of the People’s Republic of China. Then, we matched the respondents’ location information (the city and province where the participants lived during the pandemic, reported in the questionnaire) with the corresponding information of confirmed cases and finally calculated the normalized mean value of the province and city indicators as the independent variable of COVID-19 severity.

Perceived stress and individuals’ irrational buying tendency were measured through an online questionnaire, which was conducted at the end of May 2020. The questionnaire mainly contained four parts, in which respondents were asked to report on (1) their stress levels during the COVID-19 period using the updated 10-item version [49] of the original Perceived Stress Scale [10], which asks respondents to answer questions such as “how often do you feel troubled by unexpected things”, on a five-point scale (1: never; 5: all the time; α = 0.765); (2) their irrational consumption tendency during the COVID-19 pandemic on a nine-item Buying Impulsiveness Scale [50]—e.g., “I buy things without thinking” (5-point scale; 1: completely inconsistent, 5: completely consistent; α = 0.823); (3) their emotional status using the Positive and Negative Affect Schedule (PANAS) [51] (α = 0.823), and (4) their sleep quality via a nine-item scale [52], e.g., “During the COVID-19 period, how often do you suffer from the following disturbances while sleeping?”—“Cannot fall asleep within 30 min” (1: never, 2: less than once a week, 3: 1–2 times a week, 4: 3 times a week or more; α = 0.810). Importantly, these four scales were modified to ask individuals to report on their behaviors and feelings during the COVID-19 period—i.e., “Please indicate the extent to which the description below match your real situation during the COVID-19 period.” Finally, the respondents reported their demographic and economic information such as gender, age, monthly income, and education level. This study was carried out in accordance with the principles and guidelines of the declaration of Helsinki and was approved by the Local Institutional Review Boards of School of Management, Zhejiang University. All respondents gave their consent before filling in the questionnaire.

All of the statistical analyses in Study 1 were conducted using the Statistical Product Service Solutions (SPSS; Version 25.0; https://www.ibm.com/analytics/spss-statistics-software, accessed on 8 April 2022). We calculated the mean and standard deviation (SD) for all variables. A partial correlation analysis was used to preliminarily test the relationship between the severity of the COVID-19 pandemic, perceived stress, and irrational consumption. The impact of COVID-19 severity on irrational consumption was further confirmed using the hierarchical multiple regression. Finally, the mediating effect was assessed using bootstrap methods via PROCESS macro for SPSS (v3.5; A.F. Hayes; http://www.processmacro.org, accessed on 8 April 2022).

First, we matched the number of patients diagnosed with COVID-19 for each city (ranging from 4 to 50,340 persons, M ± SD = 1070 ± 6461) and province (ranging from 147 to 68,135 persons, M ± SD = 3098 ± 11,617) with respondents’ reported location and calculated the normalized mean value of the two indicators of province and city as the COVID-19 severity (ranging from 0.001 to 1, M ± SD = 0.03 ± 0.14) for each respondent.

Then, we calculated the relations among the COVID-19 severity, perceived stress, and irrational consumption tendency of the respondents. While considering covariates, including gender, age, monthly income, and education, the partial correlation analysis revealed significant positive correlations between the COVID-19 severity and the respondents’ rating scores on perceived stress (r = 0.123, *p* = 0.012) as well as their irrational consumption tendency (r = 0.120, *p* = 0.014). Consistently, after controlling for the same covariates, the hierarchical multiple regression results revealed that the COVID-19 severity positively predicted respondents’ perceived stress (adjusted R^2^ = 0.026, beta = 0.123, *p* = 0.012) and the irrational consumption tendency (adjusted R^2^ = 0.017, beta = 0.120, *p* = 0.014). In addition, the respondents’ perceived stress positively predicted their irrational consumption tendency (adjusted R^2^ = 0.061, beta = 0.245, *p* = 0.000).

**Figure 2:**
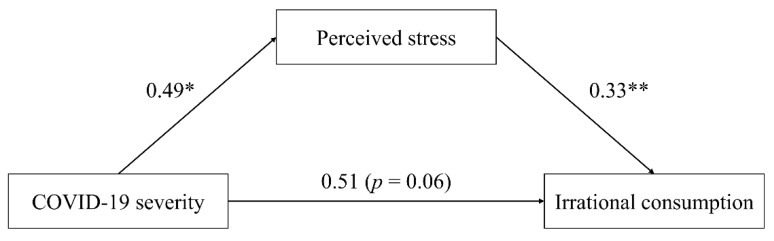
Mediation analysis: perceived stress as a mediator (Study 1). * represents significance at the 0.05 level; ** represents significance at the 0.01 level.

Consistent with our hypotheses, the mediation analysis, using Model 4 in PROCESS macro for SPSS v3.5, further revealed that the respondents’ perceived stress fully mediated the relation between the COVID-19 severity and the irrational consumption tendency (direct effect: effect = 0.515, [LLCI, ULCI] = [−0.012, 1.041]; indirect effect: effect = 0.161, [LLCI, ULCI] = [0.052, 0.297], with gender, age, monthly income, and education as covaries). Figure 2 illustrates the detailed results and confirmed that the COVID-19 pandemic is a source of stress and may lead to irrational consumption.

**Figure 3:**
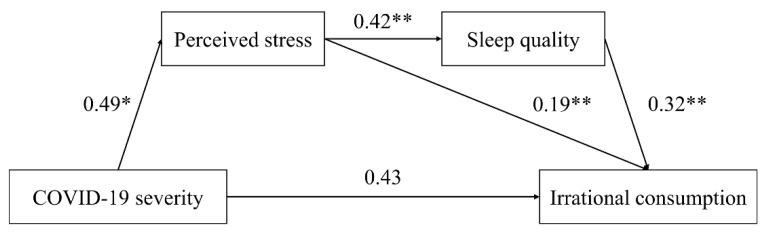
Mediation analysis: perceived stress and sleep quality as mediators (Study 1, higher scores of sleep quality scale indicates worse the sleep quality). * represents significance at the 0.05 level; ** represents significance at the 0.01 level.

We further examined the role of sleep quality in the above effect. Consistent with our hypothesis focusing on sleep quality, the chain mediation analysis via Model 6 in PROCESS macro for SPSS v3.5 revealed that perceived stress and sleep quality mediated the relation between the COVID-19 severity and the irrational consumption tendency (indirect effect: effect = 0.067, [LLCI, ULCI] = [0.022, 0.153]; with gender, age, monthly income, and education level as covariates, as shown in Figure 3). In addition, the perceived stress could also directly mediate the relation between the severity of the COVID-19 pandemic and the irrational consumption tendency (direct effect: effect = 0.435, [LLCI, ULCI] = [−0.824, 0.951]; indirect effect: stress: effect = 0.094, [LLCI, ULCI] = [0.022, 0.239]; sleep quality: effect = 0.800, [LLCI, ULCI] = [−0.065, 0.254]). This result suggests that the COVID-19 pandemic may induce increased stress and impair individuals’ sleep quality, which might further promote their irrational consumption tendency.

We also examined the potential mediating role of negative emotions using the same chain mediation analysis via Model 6 in PROCESS macro for SPSS and revealed the full mediating roles of stress and negative emotion (direct effect: effect = 0.355, [LLCI, ULCI] = [−0.155, 0.866]; indirect effect: stress: effect = 0.063, [LLCI, ULCI] = [−0.003, 0.189]; negative emotion: effect = 0.159, [LLCI, ULCI] = [−0.076, 0.368]; stress and negative emotion: effect = 0.098, [LLCI, ULCI] = [0.033, 0.208]).

These results suggest that increased stress induced by the COVID-19 pandemic may impact both individuals’ sleep quality and negative emotion and, in turn, may lead to an increase of irrational consumption tendency.

## 5. Study 2

Study 2 was then designed for two purposes: One was to replicate the impact of COVID-19 severity on perceived stress and irrational consumption tendency among consumers with different cultural background. The second was to confirm the potential underlying mechanism. As revealed above, there may be two possibilities for irrational consumption in stressful environments, relating to negative emotion and sleep quality.

The same power analysis was conducted. When the effect size was 0.15, the significance level was 0.05, and 7 predictors were arranged; the total sample size should be more than 153. Finally, 211 Western respondents (male = 69, female = 142; age = 31.80 ± 8.58; monthly income = 2856.20 ± 4589.43 US dollars) were recruited via the online platform Prolific with two requirements regarding age (required range of 18–50 years) and location (either Europe or the US) in early June 2020. The participants came from 167 cities in different countries (Europe = 180; US = 31; all respondents were proficient in English), and thus, as the COVID-19 situation impacted different areas at different degrees and time-points, this selection was expected to give a considerable range of reported severity.

The questionnaire was the same as that used in Study 1 (measuring perceived stress, α = 0.856; irrational consumption tendency, α = 0.917; negative emotion, α = 0.885; and sleep quality, α = 0.733), with an additional item examining respondents’ perceived severity of the COVID-19 pandemic in their city/town on a five-point scale (1: not severe at all; 5: extremely severe)—“How severe is the COVID-19 situation in the city you live”, which was conducted due to the unavailability of concrete numbers of infected cases in European and US cities/towns. The study was carried out in accordance with the principles and guidelines of the declaration of Helsinki and was approved by the Local Institutional Review Boards of School of Management, Zhejiang University. All respondents gave their consent before filling in the questionnaire.

The same software of SPSS and analysis methods as those used in Study 1 were used. We first examined the impact of the COVID-19 severity on perceived stress and irrational consumption tendencies via the hierarchical multiple regression, with gender, age, monthly income, and education level as covariates. The analysis showed a positive relation between the rating scores on severity of the COVID-19 pandemic (M = 2.60, SD = 1.06) and perceived stress (M = 2.73, SD = 0.39; R^2^ = 0.130, beta = 0.084, *p* = 0.001). The same regression analysis also revealed that the perceived stress positively predicted individuals’ irrational consumption tendency (M = 2.57, SD = 0.92; R^2^ = 0.106, beta = 0.371, *p* = 0.022). These results again tentatively suggest that the COVID-19 pandemic may serve as a stress source increasing individuals’ perceived stress, which may further impact individuals’ irrational consumption tendency.

**Figure 4:**
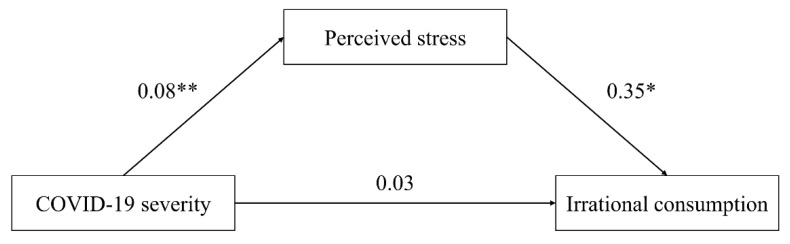
Mediation analysis: perceived stress as a mediator (Study 2). * represents significance at the 0.05 level; ** represents significance at the 0.01 level.

The mediation analysis via Model 4 in PROCESS macro for SPSS v3.5 confirmed that the respondents’ perceived stress fully mediated the relation between the COVID-19 severity and the irrational consumption tendency (direct effect: effect = 0.030, [LLCI, ULCI] = [−0.088, 0.148]; indirect effect: effect = 0.030, [LLCI, ULCI] = [0.004, 0.070]; with gender, age, monthly income, and education level as covariates). Figure 4 illustrates the detailed results.

**Figure 5:**
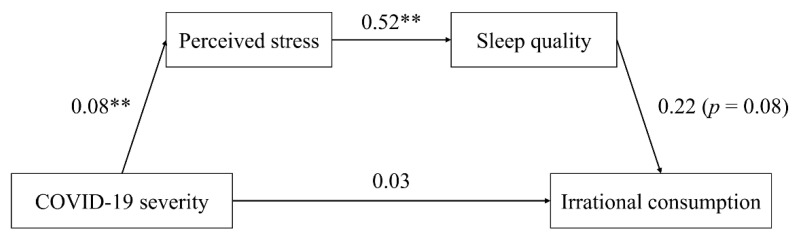
Mediation analysis: perceived stress and sleep quality as mediators (Study 2, higher scores of sleep quality scale indicates worse the sleep quality). ** represents significance at the 0.01 level.

Second, we explored the mediating role of sleep quality (M = 2.10, SD = 0.55). Consistent with the findings of Study 1, the chain mediation analysis via Model 6 in PROCESS macro for SPSS v3.5 revealed that the perceived stress and sleep quality fully mediated the relation between the COVID-19 severity and the irrational consumption tendency (direct effect: effect = 0.031, [LLCI, ULCI] = [−0.087, 0.148]; indirect effect: stress: effect = 0.020, [LLCI, ULCI] = [−0.007, 0.059]; sleep quality: effect = −0.001, [LLCI, ULCI] = [−0.025, 0.016]; stress and sleep quality: effect = 0.009, [LLCI, ULCI] = [0.001, 0.025]; with gender, age, monthly income, and education level as covariates, as shown in Figure 5). This result suggests that the COVID-19 pandemic may lead to increased stress level and impair individuals’ sleep quality, which further might promote their irrational consumption tendency.

The potential mediating role of negative emotion was also assessed; however, when considering the perceived stress and negative emotion as mediators, the chain mediation analysis via Model 6 was not significant (direct effect: effect = 0.033, [LLCI, ULCI] = [−0.085, 0.151]; indirect effect: stress: effect = 0.042, [LLCI, ULCI] = [0.007, 0.096]; negative emotion: effect = −0.004, [LLCI, ULCI] = [−0.032, 0.004]; stress and negative emotion: effect = −0.012, [LLCI, ULCI] = [−0.042, 0.007]).

## 6. Discussion

This research aimed to tentatively examine the impact of the COVID-19 severity on consumption behavior during the pandemic and to uncover the underlying mechanisms. A pilot study and two online survey studies revealed that the COVID-19 pandemic may induce increased stress, which may further impair individuals’ sleep quality and, in turn, impels irrational consumption. This research contributes to the literature of irrational buying, especially in prolonged stressful environments such as the COVID-19 pandemic, highlighting the critical role of sleep quality. Since the Chinese and the European/US cohorts showed different results in terms of negative emotion, more studies are needed to confirm the role of negative emotion in people’s consumption behavior during the prolonged COVID-19 pandemic.

Li et al. [53] demonstrated that the more serious the pandemic is, the more likely people tend to impulsively consume, which was mediated by both perceived control and materialism. Our work, focusing on individuals’ physical and mental health, adds to the existing literature that the pandemic may induce perceived stress, which further leads to irrational consumption. Moreover, we shed light on the potential mechanism underlying the stress-driven irrational consumption. That is, the increased stress during the COVID-19 pandemic may impair individuals’ sleep quality, which promotes their irrational consumption tendency.

Practically, our results have some implications for consumers and companies alike. Although shopping is a viable coping strategy to relieve consumers from stress during the COVID-19 pandemic, irrational consumption may also lead to a series of negative outcomes not only harming consumers’ financial well-being but also threatening a healthy market development. Based on our results, consumers should positively and effectively deal with the pandemic and the stress. That is, consumers could actively improve their sleep quality and mood to reduce unhealthy irrational consumption. On the other hand, companies need to pay more attention to consumers’ psychological status and their consumption during the pandemic. When the risks of infection are likely to increase, companies could boost inventory in order to prevent commodity shortages caused by irrational consumption. In addition, with economic development as well as the experience of the COVID-19 pandemic, individuals are paying more attention to their mental and physical health. Companies in the health industry should identify changes in consumers’ lifestyles and further promote consumers’ well-being.

An important topic in future research may be exploring how to reduce the adverse physical and mental consequences caused by external stressful environments and, relatedly, what activities might be better solutions than eating junk food, drinking, or making impulsive purchases. How can companies alter their presentations in order to provide a more stress-free environment that simultaneously mitigates negative impulsive behavior? Considering the relation between sleep quality and irrational buying, it is also an interesting topic to examine the effects of physiological factors, such as biorhythms, on irrational buying.

Last, this paper of course comes with limitations. The study was designed to quickly collect data from individuals in the midst of an ongoing pandemic period, and thus, there are many aspects that we did not or could not assess in light of the need for timely collection. First, we did not measure individuals’ real irrational buying behaviors due to the measures implemented to control the pandemic, even though this pilot study preliminarily confirmed that the COVID-19 pandemic might induce irrational consumption using second-hand data of the number of inter-city packages. Second, although we collected data from approximately 633 respondents and controlled for their gender, age, monthly income, and education level in Studies 1 and 2, the sample size was still not big enough to analyze more detailed mechanisms for different types of consumers, which could be carried out in future studies. That being said, it is hoped that this study would provide a preliminary insight into the current COVID-19 period, calling for attention to the mental stress issue and its impacts on individuals’ sleep quality and irrational consumption. Third, the assessments of perceived stress and irrational consumption tendency during the COVID-19 pandemic were based on retrospective methods, which limited the accuracy of the results. In future studies, objective measurement techniques, such as physiological measurements and real behavioral measurements [54], could be used in more well-designed experiment to confirm the relations between stress, sleep quality, negative emotions and irrational consumption. Fourth, the assessments of perceived severity of COVID-19 pandemic and stress level in Study 2 depended on individuals’ self-report. However, the subjective rating of the severity of the pandemic may be confounded by stress. For example, individuals very stressed by COVID-19 may rate the severity of the pandemic as more severe, and vice versa. Future studies could analyze public data on social media platform using textual analysis techniques to examine the severity of the pandemic and its impact on individuals’ psychological health and daily behaviors.

## 7. Conclusions

The current research aimed to tentatively explore the impact of the COVID-19 pandemic on individuals’ irrational consumption, revealing potential mediating roles of perceived stress as well as sleep quality. Although this study has several limitations, the findings provide preliminary evidence that the COVID-19 pandemic may induce increased stress and impair individuals’ sleep quality, which in turn might promote their irrational consumption. This research calls for attention to individuals’ physical and mental health and its negative impacts on daily activities such as consumer behavior in prolonged stressful environments.

## Data Availability

The data presented in this study are available from the corresponding author upon request. The data are not publicly available due to the requirement of approval from the Institutional Review Board.
